# Inaccurate Assessment of Canine Body Condition Score, Bodyweight, and Pet Food Labels: A Potential Cause of Inaccurate Feeding

**DOI:** 10.3390/vetsci4020030

**Published:** 2017-06-09

**Authors:** Philippa S. Yam, Gregory Naughton, Christina F. Butowski, Amanda L. Root

**Affiliations:** School of Veterinary Medicine, College of Medical, Veterinary and Life Sciences, University of Glasgow, 464 Bearsden Road, Glasgow G61 1QH, UK; gregory_n@hotmail.com (G.N.); cbutowski@hotmail.com (C.F.B.); amandaroot14@gmail.com (A.L.R.)

**Keywords:** canine, pet food labels, pet food, obesity, body condition score, body weight

## Abstract

The objectives were to investigate owners’ ability to assign the correct bodyweight (BW) and body condition score (BCS) to their dog and to interpret wet and dry pet food labels by estimating how much to feed daily. One hundred and seventy-four questionnaires were completed. Owner estimated BW was compared to actual BW, correct being defined within ±10% of actual BW. Correct interpretation of the total amount of food required was determined by the number of cans (±25% of cans) required for wet food and grams (±20% of grams) for dry food, based on the dog’s actual BW, the feeding guidelines on the label, and a comparison with the owner’s estimate. Eleven percent of owners overestimated BCS and 19% overestimated BW. Only 48% of owners could correctly estimate their dog’s BW. Only 23% and 43% of owners could correctly estimate how much wet and dry food to feed, respectively. Chi-square analysis demonstrated a significant positive association for owners correctly estimating their dog’s BW and interpreting the wet pet food label. Many owners are not aware of their pet’s BCS and BW and cannot accurately interpret pet food labels. Further owner education to improve these skills is needed if dogs are to be fed correctly.

## 1. Introduction

In the UK, 88% of owners choose a commercially prepared pet food with wet and dry pet food diets accounting for 41% and 59% of sales of commercial food, respectively [1,2]. Similar figures are published in the USA and Australia [3]. All commercial pet foods sold in the UK must state various pieces of information on the label (in accordance with FEDIAF code of practice to ensure that they comply with EU legislation) including feeding guidelines—‘The instructions for proper use should indicate how to provide a daily ration in terms of amounts required to be fed relative to the life stage, the life-style and the size of the pet’ [4]. Nevertheless, a recent survey of 8000 households claims that only 10% of dog owners read the labels on pet food [2].

It has been estimated that over half of dogs in the UK, as well as in other parts of the world, are overweight or obese [5,6,7]. This is of significance as evidence suggests that overweight and obese animals are predisposed to a number of health conditions, lifespan may be reduced, and health-related quality of life compromised [8,9,10,11]. Understanding why so many dogs are overweight and obese is crucial if it is to be tackled. In this regard, numerous papers have been published focusing on both modifiable and non-modifiable predisposing factors [12] and inaccurate feeding can be considered one such factor.

It is conceivable that failure to feed appropriate amounts of pet food may occur due to either lack of ability of owners to estimate their pet’s body condition score (BCS), bodyweight (BW), or difficulty in interpretation of the feeding guidelines. The aim of the current study was to investigate this hypothesis, and assess whether owners can estimate their pets’ BCS, BW, and the amount of food appropriate for their pet by interpreting a dry or wet pet food label.

## 2. Materials and Methods

### 2.1. Sample Population

Dogs were recruited from The People’s Dispensary for Sick Animals (PDSA) PetCheck and microchipping vehicles in four locations—Glasgow, Paisley, Dundee, and Liverpool—and from parks in Glasgow during the summers of 2014 (by GN) and 2015 (by CFB). The PDSA PetCheck vehicles provide a free service open to all dog owning members of the general public, and the organization aims to provide general council on the health and welfare of pet animals. Owners were approached and asked to fill in a paper-based questionnaire and were recruited solely on their willingness to participate with no exclusions applied. All participants gave consent and approval for this study was granted by the University of Glasgow, School of Veterinary Medicine, Ethics and Welfare Committee.

### 2.2. Data Collection

BCS was assessed using a 5-point scale [13]. 1: emaciated, 2: lean, 3: ideal, 4: overweight, 5: obese. The dogs were weighed on a set of scales (Marsden Group). All body condition scoring and weighing of the dogs was completed in 2014 by GN and in 2015 by CB, who were trained by PY who has extensive experience in this procedure. The interviewer’s assessment of BCS and BW of each dog was taken as the gold standard.

### 2.3. Questionnaire and Assessment of Pet Food Labels

The questionnaire was paper-based and designed at the University of Glasgow (Supplementary Material). It is comprised of four sections: Section 1 asks general questions about the dog including BCS (in both word and picture format) and BW as estimated by the owner. Section 2 asks about the general health and wellbeing of the dog. Section 3 asks the owners to read and interpret a wet pet food label (wet pet food label used was from a 400 g tin of Pedigree complete adult original dog food formula) (2014 and 2015) and a dry pet food label (dry pet food label used was from a 5 kg box of Purina Bakers complete adult dog food formula) (2015) to determine how much of that brand of food they would feed their dog in total in a day if fed exclusively (the question asked was, “If you were feeding just this food to your dog (i.e., no mixer or table scraps), how many grams or tins would you give in total each day?”). Section 4 was used to record the actual BCS and BW of the dog (as assessed by GN and CB).

### 2.4. Statistical Methods

Data from both collection periods were amalgamated and stored in an Excel spreadsheet (Microsoft Office 2011). Only questionnaires that were completed in full were analysed. Owners were asked to allocate a BCS of their dog in picture and word format. Each of these answers was then compared to the actual BCS and assigned a score of 1 (correct) or 0 (incorrect). Owner estimated BW was compared to actual BW, correct being defined as within ±10% of actual BW. All the dogs were given a breed category; however, to account for the dogs that were not purebred, a category of crossbreed was included. For each specific breed, a single category was made and listed as such.

Microsoft Excel (Analysis-Tool Kit for Excel 2016) and Minitab v.17 (Minitab Inc., State College, PA, USA) were used to generate frequencies, prevalence, descriptive statistics, distribution, and chi-square tests. Descriptive statistics were done on the dogs’ age, weight, actual BCS, desired BCS, population BCS, and estimated BCS (word and picture). Statistical significance of the variables was tested using chi-square analysis, which was performed on the quantitative data, BCS and BW, for all breed groups (crossbreeds, pedigrees, and all dogs). The prevalence of misperception was calculated as the proportion of owners who could correctly identify their dog’s BCS and BW. The information from the open and closed-ended questions were analysed quantitatively, and themes were identified.

Correct interpretation of the total amount of wet food required to feed their dog in one day was assessed by determining the number of cans required, based on the dog’s actual BW and the feeding guidelines on the label, and then comparing it to the owners estimate (correct to within ±25% of cans recommended for actual BW). Correct interpretation of the total amount of dry food required to feed their dog in one day was assessed by determining the number of grams required, based on the dog’s actual BW and feeding guidelines on the label, and then comparing it to the owner’s estimate (correct to within ±20% of grams recommended on the label, increments being calculated for each kg of BW). Each of these parameters was then compared using the chi-square test, correct interpretation of a wet or dry pet food label compared to actual BW. Statistical significance was defined as *p* < 0.05.

## 3. Results

Over the sampling period, a total of 189 questionnaires were completed; 89 in 2014, and 100 in 2015. Of these, 15 had missing information providing 174 fully completed questionnaires for further analysis.

The signalment of the dogs included in the analysis are listed in Table 1. The actual BCS were BCS 2, 17% (n = 29); BCS 3, 45% (n = 79); BCS 4, 31% (n = 54), and BCS 5, 7% (n = 12). West Highland White Terriers (*p* < 0.01; *p* < 0.02) and Cocker Spaniels (*p* < 0.03; *p* < 0.04) were the only breeds to be overweight or obese compared to any other pedigree or crossbreed dog, respectively.

**Figure 1:**
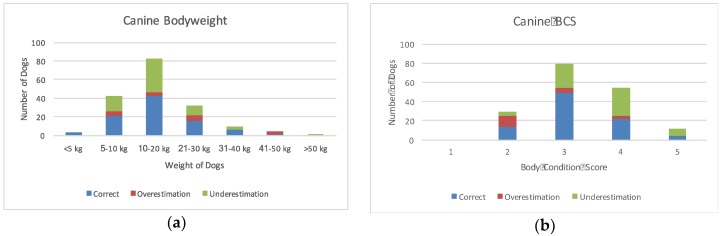
Owner assessment of canine (**a**) bodyweight in kilograms and (**b**) body condition score on a five-point scale. Bars are divided in owner misperception types (correct estimation, overestimation and underestimation).

Just over half of the owners were able to correctly identify their dog’s BCS, either by word association (55%) or by picture (51%) (Table 2). Nineteen owners (11%) overestimated their dog’s BCS and 66 owners (38%) underestimated their dogs BCS—50 owners by 1 BCS increment, 13 by 2 BCS increments, and 3 by 3 BCS increments. Only 48% of owners were able to correctly estimate their dog’s BW; of those that got it wrong, 33% underestimated and 19% overestimated the weight of their dog by greater than 10% of actual BW (Figure 1).

Forty-nine percent of owners could correctly quantify how much wet food to feed by interpretation of the wet food label, 44% of owners underestimated, and 7% overestimated. For the dry food, 44% of owners correctly quantified, 42% underestimated, and 14% overestimated how much to feed by interpretation of the dry food label. Chi-square analysis demonstrated a significant positive association (*p* = 0.01) for owners correctly estimating their dog’s BW and interpreting the wet pet food label, but there was no statistically significant association between the estimate of BW and dry food requirement (Table 3).

## 4. Discussion

The Pet Food Manufacturers Association estimates that 88% of UK owners choose a commercially prepared pet food [2] and advise owners to follow feeding guidelines, adapting to the individual needs of their pet. Feeding guidelines are a vital piece of information on the pet food label as they provide recommended portion sizes based on size/weight of a pet. Therefore, owner ability to accurately assess BCS and BW and interpret pet food feeding guidelines is important for the correct provision of calorie requirements. The current study demonstrated that in a cohort of randomly selected dog owners, just over half could accurately assess their dog’s BCS and of those that got it wrong, 78% underestimated it. These findings of misperception of body shape substantiate those previously reported and owner underestimation of BCS has been shown to be most prevalent in owners of obese dogs and is a risk factor of obesity [14,15,16]. The current study also concurs with previous findings in that owners struggle to correctly identify BCS even with the aid of pictures [17], so the use of these images on pet food labels will only be useful if owners are educated on how to interpret them correctly.

Overall, we found that 38% of dogs were either overweight or obese. In the most recently published canine studies, between 16 and 34% of dogs were found to be overweight [18,19] and between 33 and 41% obese globally [20], which is in line with our current findings. Owners need to be educated about modifying feeding rations if their pet’s BCS is not within an ideal range. Based on these findings, investigating whether owners are aware of this is justified as part of a future study. Furthermore, less than half of owners were able to correctly estimate BW within 10% accuracy. Although there was a significant association between owner estimation of BW and interpretation of the wet pet food label, this was not the case for the dry label, and this is a concern given that most owners in the UK feed dry food to their dogs [1].

Owner motivation to read pet food labels may correlate with how motivated they are to read and acknowledge human food labels [21]. If owners neither recognise that their pets are underweight or overweight, nor realise the dangers of each, and if they are not used to looking at labels on food for their own consumption and have difficulty interpreting pet food labels, they may not in turn regulate the amount of food they provide to their pet appropriately. The ramification of inaccurate feeding is that owners could be either inadvertently providing excess calories to their pets, resulting in overweight and obesity, or providing insufficient calories, resulting in malnutrition. Balanced nutrition ensuring adequate intakes of energy, protein, minerals, and vitamins is essential for dogs to ensure health and longevity [22]. An interesting finding from this study was that many owners seemed to underestimate the amount that their dogs should be fed—for both dry and wet food. Given the high prevalence of overweight and obesity in the study population, this was a surprising finding. One can speculate the reasons for this; it may be that owners who underestimated how much to feed based on the pet food labels may be providing calories to their dogs by other means such as in the form of table scraps and treats; estimates suggest 53% of owners who feed table scraps do so daily [2]. Owners may be feeding a combination of dry and wet pet foods, thus incurring errors in quantity required or may have adjusted the quantity for their dog’s energy level (up to 50% more for active dogs or up to 20% less for less active dogs) as stated on the wet pet food label even though it was not specifically stated on the questionnaire. Alternatively, owners are not measuring the food correctly, or the pets are not expending sufficient calories for the amount of food given.

Further causes of error in energy intake can result from varying energy allowances, recommended for maintenance of adult dogs; current label recommendations are an inexact science. Brands may use different energy maintenance requirement formulas to guide feeding instructions, causing variability in energy content, as figures ranging from less than 90 kcal ME/kg^0.75^ (377 kJ) to approximately 200 kcal ME/kg^0.75^ (810 kJ) have been published for adult maintenance [23]. Furthermore, the method by which the energy content of food is determined might be important, namely whether measured in feeding trials, or calculated and, if so, by what method, modified Atwater factors [24] or NRC guidelines [25]. Calculations based upon modified Atwater factors often under-estimate the actual energy content of commercial foods of dogs [26], perhaps accounting for the current propensity for canine obesity. The cause of inaccurate feeding is complex and owners should be advised about appropriate levels of feeding and the modification required for underweight or overweight pets.

A significant association was found between wet food label interpretation and correct BW estimation. This may be due to canned pet food having less exact feeding guidelines (halves and thirds of a can used as guidelines) compared to dry food. The feeding of dry food may be complicated further since, even if owners do interpret the label correctly, they typically underestimate the amount of food they are providing when using feeding cups [27]. Hence, even if owners could interpret the label, the dogs may still be overfed. The same argument could be applied to the large numbers of owners, who also underestimated how much dry food to feed, as they too may ultimately end up overfeeding. For both reasons, namely an inability to interpret the dry food label and imprecision when using feeding cups, recommendations to weigh the appropriate amount of dry food for pets is important to accurately feed overweight dogs. Dry pet food labels may have been more difficult to interpret for a few reasons, one being that the dry pet food label lists broad ranges of weight categories and grams per feed. Grams per feed differences ranged from 85 to 225 g. Secondly, the open-ended, quantitative question for the dry food (blank with number of grams to be written by the owner) may have confused owners in comparison to the wet food label, where the options were listed out for them (specific categories of fractions of cans) to choose. This may help to explain a difference in the accuracy of comparing the wet and dry pet food labels.

Whilst the aim of our study was fulfilled and indicated that owners are inaccurate when feeding pets, the results should be interpreted with caution. A limitation of the study was that the participants were recruited from free services and may not be representative of the general population. Therefore, it would be worthwhile to pursue additional larger studies enlisting owners from a wider cross section of society and comparing the accuracy of BCS, BW, and/or feeding instruction among groups of owners with underweight, ideal and overweight dogs, or owners with varied demographics, which has been shown to influence pet obesity. It would have been interesting to ascertain whether owners were able to interpret labels once given the true weight of their pets. Additionally, the data from the two different time points (2014 and 2015) were not statistically assessed in this study, so a bias in assessments or any differences between the findings from the two collection periods cannot be ruled out. Although the owners were asked to interpret only two labels, a wet and dry pet food label, we did not establish what food the owners typically fed their pet, and additional labels were not investigated. The questionnaire could include the dog’s typical feeding regimen, thus allowing a comparison between current and novel feeding. In this regard, in future studies, a range of different labels could be investigated from both dry and wet foods to tease out further information about the presentation of data and the ease, or otherwise, of interpretation. Finally, the expected daily caloric requirement for each animal was not calculated and compared to caloric density of the dry or wet pet food. Various formulae for maintenance energy requirements [23] are available, but limited information about factors that could alter these requirements, such as physical activity and home conditions, were not known, so the crude feeding guidelines on the wet and dry food labels were followed. As feeding guidelines for dry food are suggested in “grams” and for wet food as “cans”, we designated greater than ±20% grams according to feeding guidelines for dry food and ±25% for wet food based on the feeding guidelines as correct. Other values could have been selected, but this seemed a sensible starting point by which to ascertain a general guideline of owners’ ability to estimate these measurements (particularly without any other knowledge about other factors impacting on calorie requirements of these animals being available). Further, it would have been interesting to ask the owners to actually ‘plate up’ the food they would give in a day so that this could be measured.

This study has shown that many owners are not aware of their pet’s BCS and BW and that many owners cannot accurately interpret pet food labels. Perhaps the current findings could be viewed as encouraging inasmuch as inaccurate feeding due to lack of recognition of body size and shape together with inability to interpret pet food labels are factors that can all be tackled by owner education. The current findings lend credence to the instigation of owner education about the monitoring of their pets’ BCS and BW, adjusting feeding regimes accordingly, and the interpretation of pet food labels.

## 5. Conclusions

This study used a novel questionnaire to determine how well owners could assess BCS and BW and accurately interpret wet and dry pet food labels. Analysis of the questionnaire scores showed that around half of the sample population was unable to correctly identify any of these parameters. These figures clearly indicate that more needs to be done by veterinarians and the pet food industry to educate owners on the importance of a pet’s weight, a pet’s shape, and pet food label interpretation. Veterinary surgeons need to change owner behaviour by counselling them about the importance of appropriate BW and the deleterious effects of obesity and should aim to help owners with their feeding regimes, particularly for dry food, educating them throughout their dog’s life so that they are fed to an ideal BCS for health and longevity.

## Figures and Tables

**Table 1:** Signalment of dogs included in study.

	Frequency
**SEX**	Number (%)
Male Neutered	44 (26%)
Male Entire	57 (33%)
Female Neutered	42 (24%)
Female Entire	30 (17%)
**AGE**	
0-3 years	94 (54%)
4-7 years	44 (25%)
≥ 8 years	36 (21%)
BREED *	
Bedlington Terrier	3
Bichon Frise	7
Border Collie	10
Border Terrier	5
Boxer	2
Cavalier King Charles Spaniel	3
Cocker Spaniel	11
Crossbreed	54
Golden Retriever	2
Husky	5
Jack Russell	6
Labrador	12
Lakeland Terrier	3
Lhasa Apso	2
Lurcher	2
Maltese terrier	2
Pug	5
Schnauzer	2
Shih Tzu	4
Springer Spaniel	5
Staffordshire bull terrier	3
Tibetan Terrier	3
West Highland White Terrier	8

* There was one each of the following breeds: Beagle, Cairn Terrier, Dachshund, English Bulldog, Eurasier, German Spitz, Greyhound, Hungarian Vizsla, Irish Terrier, Irish Water Spaniel, Italian Spinone, Rottweiler, Welsh Terrier, Whippet, and Yorkshire Terrier.

**Table 2:** The number and percentage of owners who correctly assessed, overestimated, and underestimated the body condition score (BCS) by word description and picture, bodyweight (BW) of their dog, and amount to feed following interpretation of wet and dry pet food labels.

	Sample Size (n=)	Correct n (%)	Overestimation n (%)	Underestimation n (%)
BCS (word)	174	96 (55%)	30 (17%)	48 (28%)
BCS (picture)	174	89 (51%)	19 (11%)	66 (38%)
BW Estimate	174	84 (48%)	33 (19%)	57 (33%)
Wet Pet Food Label	174	85 (49%)	13 (7%)	76 (44%)
Dry Pet Food Label	92	40 (44%)	13 (14%)	39 (42%)

**Table 3:** Pearson’s chi-square test for the assessment of correct interpretation of pet food labels and the correct estimation of bodyweight (BW).

Analysis	Chi-square Value	Degrees of Freedom	*p*-Value
Correct Wet Label vs. Correct BW	6.579	1	0.010
Correct Dry Label vs. Correct BW	0.005	1	0.941

## Supplementary Material

The following are available online at http://www.mdpi.com/2306-7381/4/2/30/s1, Questionnaire.
