# Supplementary material for: Inaccurate Assessment of Canine Body Condition Score, Bodyweight, and Pet Food Labels: A Potential Cause of Inaccurate Feeding
Source: Vet Sci. 2017 Jun 9;4(2):30. doi: 10.3390/vetsci4020030 (PMC5606605; doi:10.3390/vetsci4020030)
Supplement: Supplementary file 1 [file vetsci-04-00030-s001.pdf]

University of Glasgow | School of Veterinary Medicine

pdsa
for pets in need of vets

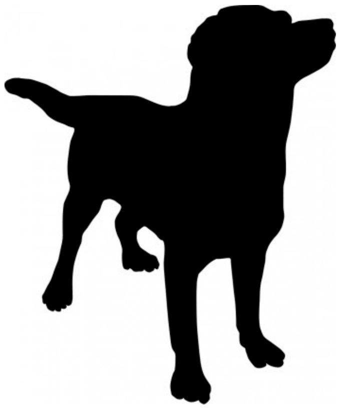

Aim of the survey

We are investigating dog body shape and pet food labels.

About the survey

- This survey contains 4 sections. It should take 5 minutes to complete.
- It asks for details about you and your dog.
- All data will be anonymous so we can't identify individuals
- Consent to take part in this study will be inferred through completion of the questionnaire

THANK YOU

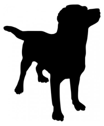

Section 1: Details about you and your dog

About your dog	
Age of your dog	________ Years ________Months
Sex of your dog	☐ Entire Male ☐ Entire Female<br>☐ Neutered Male ☐ Neutered Female
Breed	☐ Pedigree ☐ Crossbreed
If your dog is a pedigree, please write the breed in the box	
Please estimate how much your dog weighs (either in stone/lbs/kgs)?	__________________stone/lbs<br>Or<br>__________________kg
Which of the word descriptions best describes your dog's body shape?	☐ Thin ☐ Slightly thin ☐ Just right<br>☐ Slightly overweight ☐ Overweight
When you walk your dog on the average day, is he/she on or off the lead most of time?	☐ He/she is on the lead most of time<br>☐ He/she is off the lead most of the time
How much time on average does your dog spend running off lead/after toys ?	☐ Less than 10 minutes<br>☐ 10 – 30 minutes<br>☐ 30 – 60 minutes<br>☐ 60 minutes or more<br>☐ Don't know
Do you feed your dog table scraps?	☐ Never<br>☐ Every day<br>☐ A few times a week<br>☐ Less than weekly<br>☐ Never

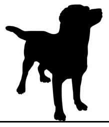

Health of your dog

Does your dog have an ongoing illness or condition?<br><br>If so please list:	☐ Yes ☐ No
Is your dog on any current medication?<br>If so please list:	☐ Yes ☐ No
When was your dog's last visit to the vet?	☐ less than 3 months ago<br>☐ between 3 months and one year ago<br>☐ over a year ago
How do you think your dog would rate his/her quality of life?'<br><br>From 0-6 where 0 is couldn't be worse to 6 being couldn't be better?	☐ ☐ ☐ ☐ ☐ ☐ ☐<br>0 1 2 3 4 5 6<br>Bad Great

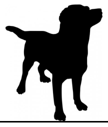

**About you**	
Your age (Years)	☐ 18-35 ☐ 36-50 ☐ 51-65<br>☐ > 65
Your gender	☐ Male ☐ Female
What is your personal annual income?	☐ Less than £10,000<br>☐ £10,000-£20,000<br>☐ £20,000-£40,000<br>☐ > £40,000<br>☐ Don't know / Would rather not say
What description best fits your education?	☐ No formal qualifications<br>☐ Standard grades/GCSE<br>☐ Higher grades/A levels<br>☐ Degree<br>☐ Postgraduate qualification<br>☐ Don't know / Would rather not say
What are the first 4 letters of your postcode?	

Section 2: Your dog's body shape

On the next page, there are some pictures of different body shapes of dogs.

Please tick the box next to the picture that best describes your dog.

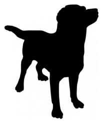

	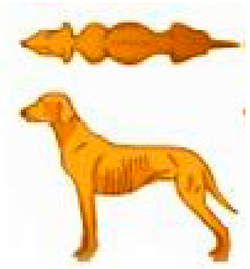	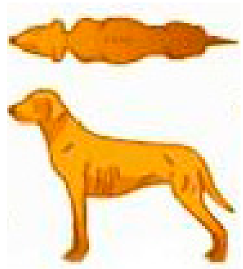	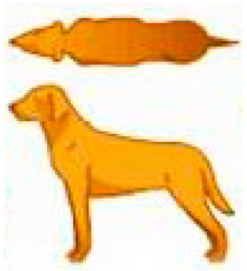	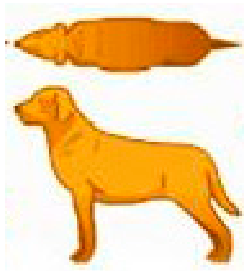	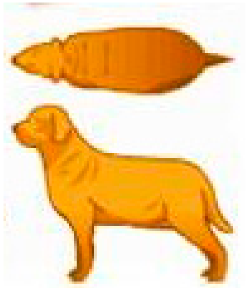
Q1: Please tick the body shape that is closest to your dog?					
Q2: Please tick the body shape that you would most like your dog to be?					
Q3: Please tick the body shape that most dogs you see regularly look like?					

Section 3: Pet food labels

Below is a label from a dog food can.

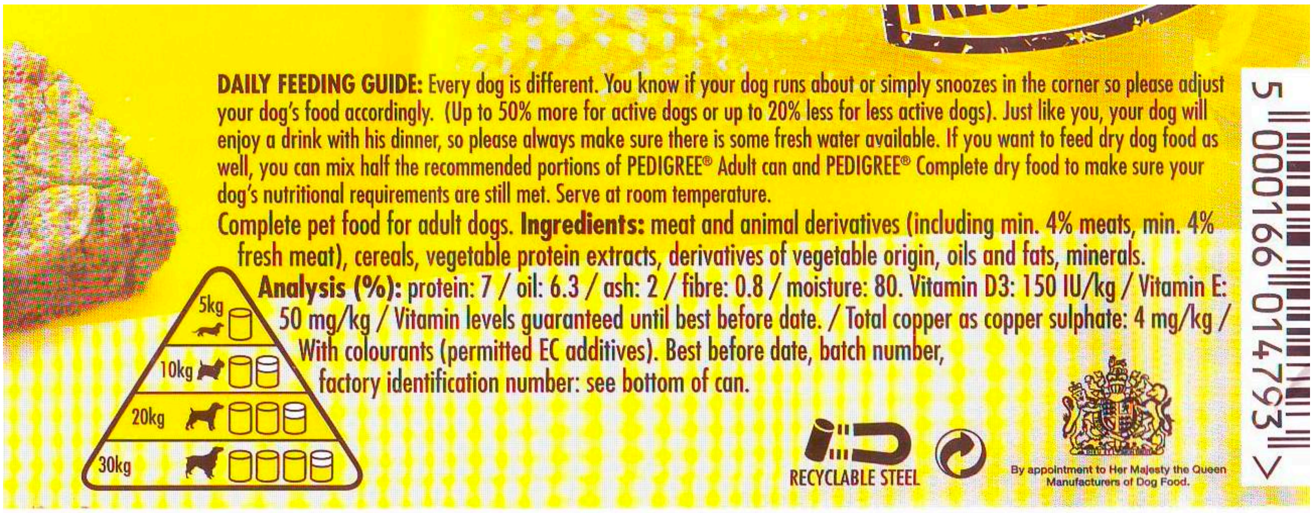

If you were feeding **just** this food to your dog (i.e. no mixer or table scraps), how many tins would you give in total each day?

- ☐ ½ tin
- ☐ 1 tin
- ☐ 1 ½ tins
- ☐ 2 tins
- ☐ 2 ½ tins
- ☐ 3 tins
- ☐ 3 ½ tins
- ☐ 4 tins
- ☐ 4 ½ tins
- ☐ 5 tins

- ☐ Other (please write in amount)_____tins

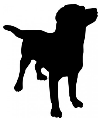

Below is a label from dry dog food.

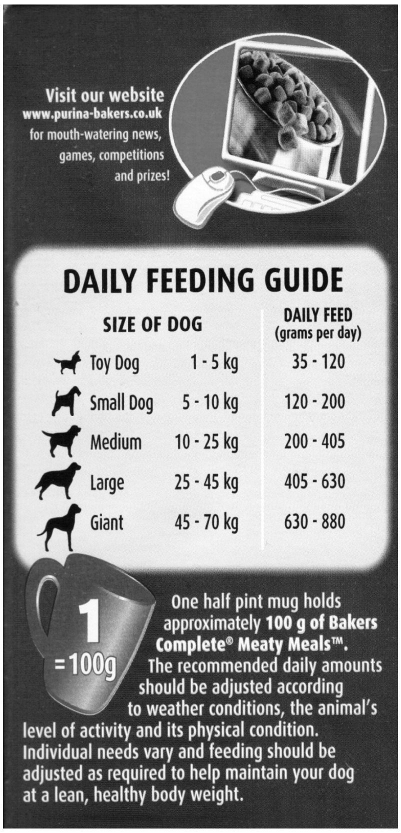

Visit our website
www.purina-bakers.co.uk
for mouth-watering news, games, competitions and prizes!

DAILY FEEDING GUIDE

SIZE OF DOG		DAILY FEED (grams per day)
Toy Dog	1 - 5 kg	35 - 120
Small Dog	5 - 10 kg	120 - 200
Medium	10 - 25 kg	200 - 405
Large	25 - 45 kg	405 - 630
Giant	45 - 70 kg	630 - 880

1 = 100g

One half pint mug holds approximately **100 g of Bakers Complete® Meaty Meals™.** The recommended daily amounts should be adjusted according to weather conditions, the animal's level of activity and its physical condition. Individual needs vary and feeding should be adjusted as required to help maintain your dog at a lean, healthy body weight.

If you were feeding just this food to your dog (i.e. no mixer or table scraps), how many grams would you give **in total** each day?

_____________________________ grams

[Type text]

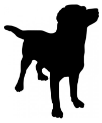

Section 4: Your dog's measurements

We will body condition score and weigh your dog. The results will be recorded on this sheet.

If you do not want to take part in this part of the study, please leave your questionnaire in the box provided.

**Your dog's measurements**	
Your dogs body condition score is	1 2 3 4 5
Your dog's weight is (kg)	
Advice given to owner	
